# Leveraging genetics to investigate causal effects of immune cell phenotypes in periodontitis: a mendelian randomization study

**DOI:** 10.3389/fgene.2024.1382270

**Published:** 2024-06-21

**Authors:** Yingjie Bai, Pengxian Xie, Ziyu Jin, Shengao Qin, Guowu Ma

**Affiliations:** ^1^ School of Stomatology, Dalian Medical University, Dalian, China; ^2^ Academician Laboratory of Immune and Oral Development and Regeneration, Dalian Medical University, Dalian, China; ^3^ School of Stomatology, North China University of Science and Technology, Tangshan, China; ^4^ International Business College, Dongbei University of Finance and Economics, Dalian, China; ^5^ Salivary Gland Disease Center and Beijing Key Laboratory of Tooth Regeneration and Function Reconstruction, Beijing Laboratory of Oral Health and Beijing Stomatological Hospital, Capital Medical University, Beijing, China; ^6^ Beijing Laboratory of Oral Health, Capital Medical University, Beijing, China; ^7^ Department of Stomatology, Stomatological Hospital Affiliated School of Stomatology of Dalian Medical University, Dalian, China

**Keywords:** Mendelian randomization, risk factor, immune system, immune cells, periodontitis

## Abstract

**Introduction:**

Immune cells are dynamic in the inflammatory environment and play a key role in eradicating periodontal pathogens, modulating immune responses, and instigating tissue destruction. Identifying specific immune cell phenotypes associated with periodontitis risk is essential for targeted immunotherapeutic interventions. However, the role of certain specific immune cell phenotypes in the development of periodontitis is unknown. Mendelian randomization offers a novel approach to reveal causality and address potential confounding factors through genetic instruments.

**Methods:**

This two-sample Mendelian randomization study assessed the causal relationship between 731 immune cell phenotypes and periodontitis using the inverse variance weighting method with the GWAS catalog genetic database. Methodological robustness was ensured through Cochran’s Q test, MR-Egger regression, MR-PRESSO, and Leave-One-Out analysis.

**Results:**

14 immune cell phenotypes showed potential positive causal associations with periodontitis risk (*p* < 0.05), suggesting an increased risk, while 11 immune cell phenotypes exhibited potential negative causal associations (*p* < 0.05), indicating a reduced risk. No significant heterogeneity or pleiotropy was observed.

**Conclusion:**

This study underscores certain immune cell types as potential periodontitis risk biomarkers, laying a theoretical foundation for future individualized treatment and precision medicine development.

## 1 Introduction

Periodontitis (PD) is an inflammatory disease caused by the imbalance of local microbial communities, which can lead to the destruction of tooth-supporting tissues (Slots, 2000). In addition, it is associated with systemic diseases and can exacerbate or trigger other systemic diseases such as rheumatoid arthritis and diabetes (González-Febles and Sanz, 2000; Chen et al., 2022). Emerging evidence suggests that PD is also related to the composition and function of the gut microbiota. The gut flora may interact with the oral flora and participate in the development of PD (Luo et al., 2023; Chen et al., 2024). It is now widely believed that the pathogenesis of periodontal disease is not only caused by infecting microorganisms, but also by over-activation of the host immune response (Loos and Van Dyke, 2000; Han et al., 2023).

Immune cells are a key component of the body’s natural defense against foreign invaders. They are made up of different subpopulations with a large number of special functions, mainly including T cells, B cells, dendritic cells, macrophages, and monocytes (Page et al., 2000). The differentiation of immune cell subsets and their complex interactions in the internal biological environment constitute a ‘pathophysiological network’ that plays a dual role in the development of inflammation (Fang et al., 2018; Margraf and Perretti, 2022; Peng et al., 2022; Chen et al., 2023). Immunophenotype, as a critical indicator of immune system activity, refers to the expression pattern of surface molecules and receptor molecules of immune cells, which can regulate processes such as cell growth, differentiation and activity. During the diagnosis of diseases such as inflammation and infection, doctors can identify different receptors and molecules on the surface of immune cells to determine the type of disease and severity of the condition for better treatment. Therefore, understanding the phenotype of immune cells is crucial for the study of various immune-related diseases, as well as for advancing therapeutic strategies.

While previous observational studies have characterized immune cell profiles in periodontal tissues, they often lack the ability to establish causal associations or identify protective versus pathogenic immune responses. In contrast, Mendelian Randomization (MR) offers a novel approach to address several gaps and limitations in existing literature on the immune response in PD. In recent years, a large number of studies have utilized MR to identify the association between immune cell phenotype and disease, providing new insights into the development of more effective treatment strategies (Aru et al., 2023; Han et al., 2024).

MR, leveraging genetic variants as instrumental variables, allows for the exploration of causal relationships between exposures and outcomes, overcoming confounding and reverse causality biases inherent in observational studies, and saves human and material resources for subsequent studies by precise target screening (Burgess et al., 2018). By utilizing MR, this study aims to elucidate the causal links between 731 immune cell phenotypes and PD. This approach represents a paradigm shift in periodontal research, offering a robust framework to discern causal associations and identify potential therapeutic targets.

## 2 Materials and methods

### 2.1 Data sources

**FIGURE 1:**
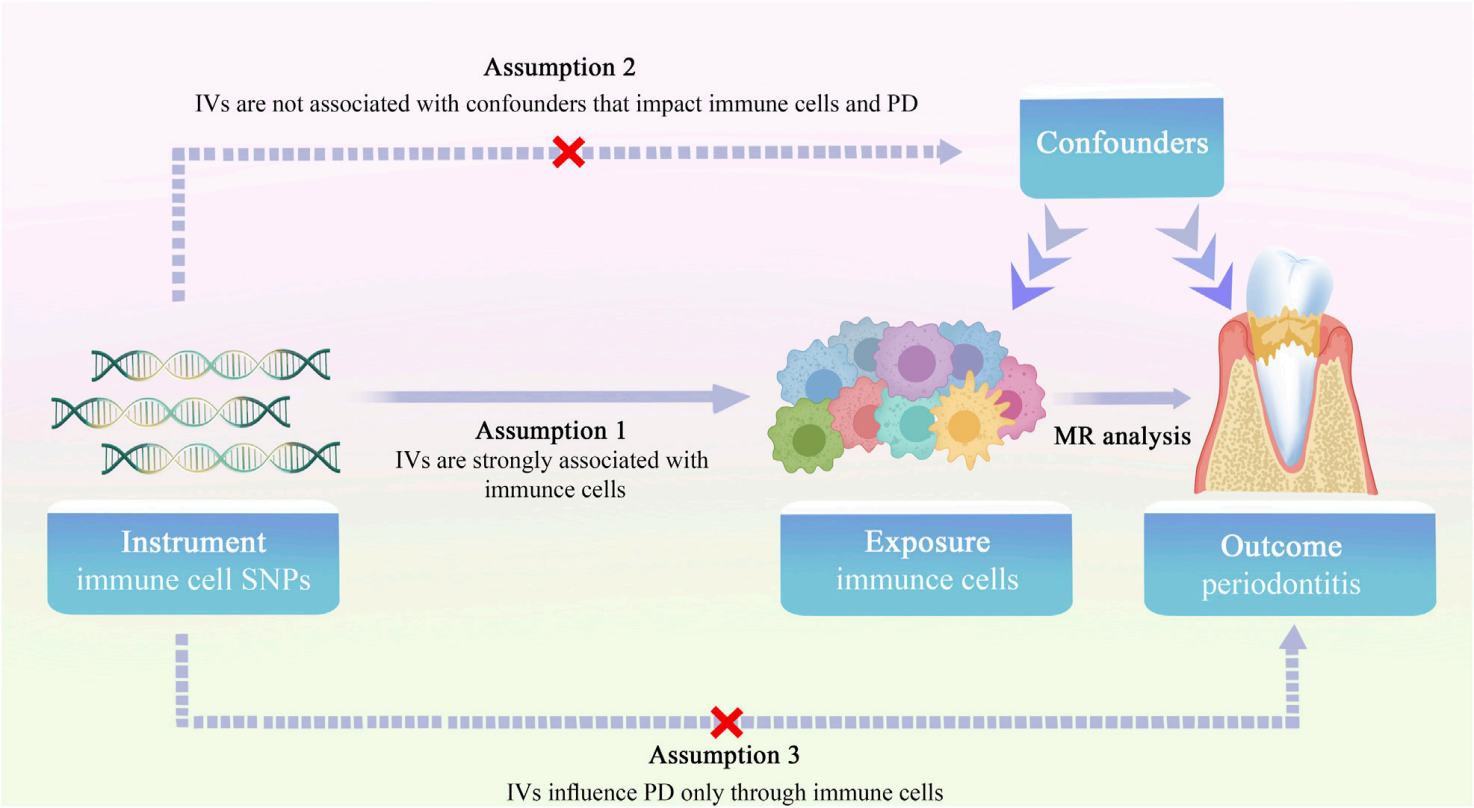
Overview of the study design for two-sample MR investigating the causal effects of immune cell phenotypes on PD.

The schematic diagram of the MR study investigating the causal effects of immune cells phenotypes on PD is shown in Figure 1. The dataset for PD in this study was obtained from the OpenGWAS database, focusing on European samples (https://gwas.mrcieu.ac.uk). The study encompassed 3,046 European adults diagnosed with PD and a control sample of 195,395 European adults, retrieved from GWAS data. Summary statistics for 731 immune traits were obtained from the GWAS catalog database, accessible through accession numbers GCST09001391 to GCST90002121 (Orrù et al., 2020). These immunophenotypes covered various parameters, including absolute cell (AC) counts (*n* = 118), median fluorescence intensity (MFI) reflecting surface antigen levels (*n* = 389), morphological parameters (MP) (*n* = 32), and relative cell (RC) counts (*n* = 192). Specifically, MFI, AC, and RC features included panels such as B-cells, conventional dendritic cells (cDCs), T-cell maturation stages, monocytes, myeloid cells, TBNK (T-cells, B-cells, natural killer cells), and regulatory T cells (Tregs). The MP feature included cDC and TBNK panels.

### 2.2 Instrumental variable selection

In the MR analysis, single nucleotide polymorphisms (SNPs) served as instrumental variables (IVs) to assess the causal relationship between immune cell traits and PD. Selection of IVs adhered to three hypotheses: (1) the correlation hypothesis, indicating a strong correlation between IVs and immune cell traits; (2) the exclusivity hypothesis, affirming no direct relationship between IVs and PD traits; and (3) the independence hypothesis, ensuring no correlation between IVs and confounding factors (Davies et al., 2018). IVs, chosen based on genome-wide significance (*p* = 1 × 10^−5^) and conditions of chain imbalance threshold (*r* ^2^ = 0.001, kb = 10,000), underwent further refinement. To exclude the effect of bias of weak IVs, the F statistic of each IV was calculated, with IVs having F < 10 being excluded, and only those with F > 10 included for MR analysis. In this study, a reverse MR analysis of PD and immune cells was performed to screen eligible IVs at a threshold of *p* = 5 × 10^−6^.

### 2.3 Statistical analysis

The evaluation of the causal relationship between immune cells and PD was primarily conducted using R software (version 4.3.2), MRPRESSO (1.0), and Two Sample MR (0.5.8). MR analysis utilized inverse variance weighting (Burgess et al., 2013), weighted median (Bowden et al., 2016) and MR-Egger methods (Bowden et al., 2015). A significant causal relationship between exposure and outcome was considered when *p* < 0.05. Heterogeneity, which refers to the variability in causal estimates across different genetic variants used as instrumental variables (IVs), was assessed using Cochran’s Q test and corresponding *p*-values. A significant *p*-value from Cochran’s Q test indicates heterogeneity in the causal estimates, suggesting that the IVs may not be valid due to underlying differences in their effects on the exposure of interest. To address potential pleiotropy or other biases that might affect the MR analysis results, we employed several sensitivity analyses: 1). MR-Egger Regression: The method can assess the presence of directional pleiotropy by testing for non-zero intercepts in the regression model. A significant intercept suggests the presence of pleiotropy, indicating that the MR estimates may be biased. 2). MR-PRESSO: It detects outlier SNPs that exhibit significant differences in the causal estimates compared to the overall trend, allowing for the correction of pleiotropy-induced bias. 3). Leave-One-Out Analysis: We conducted leave-one-out sensitivity analyses to assess the influence of individual SNPs on the MR estimates, thereby evaluating the robustness of the overall findings. 4). Funnel Plots: We generated funnel plots to visually inspect for asymmetry, which may indicate the presence of pleiotropy or other biases in the MR analysis.

## 3 Results

### 3.1 Instrumental variable result

In this study, the exposure factors included 731 immune cell phenotypes. A total of 559 SNPS were included in the two-sample MR analysis based on the SNPS screening criteria.

### 3.2 Causal relationship between immune cells and PD

**FIGURE 2:**
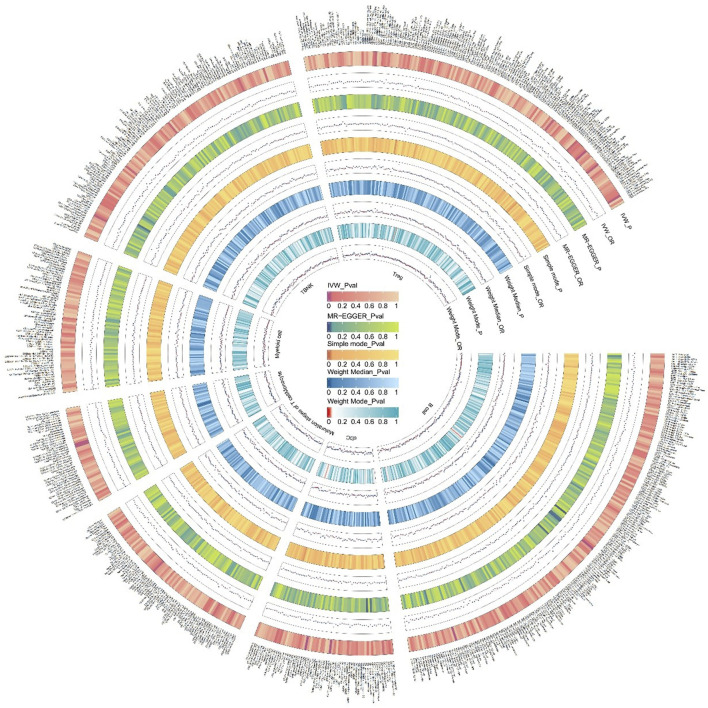
We conducted an initial MR analysis to investigate the relationship between 731 immune cells and the risk of PD. The various circles in the figure denote different methods used to estimate the results, comprising inverse variance weighting (IVW), MR-Egger, Simple mode, Weight Median, and Weight Mode. Immune cells were classified into distinct categories, including T cells, B cells, myeloid cells, monocytes, cDCs, T cell maturation stage, TBNK, and other categories. The color shade of each circle corresponds to the magnitude of the *p*-value.

**FIGURE 3:**
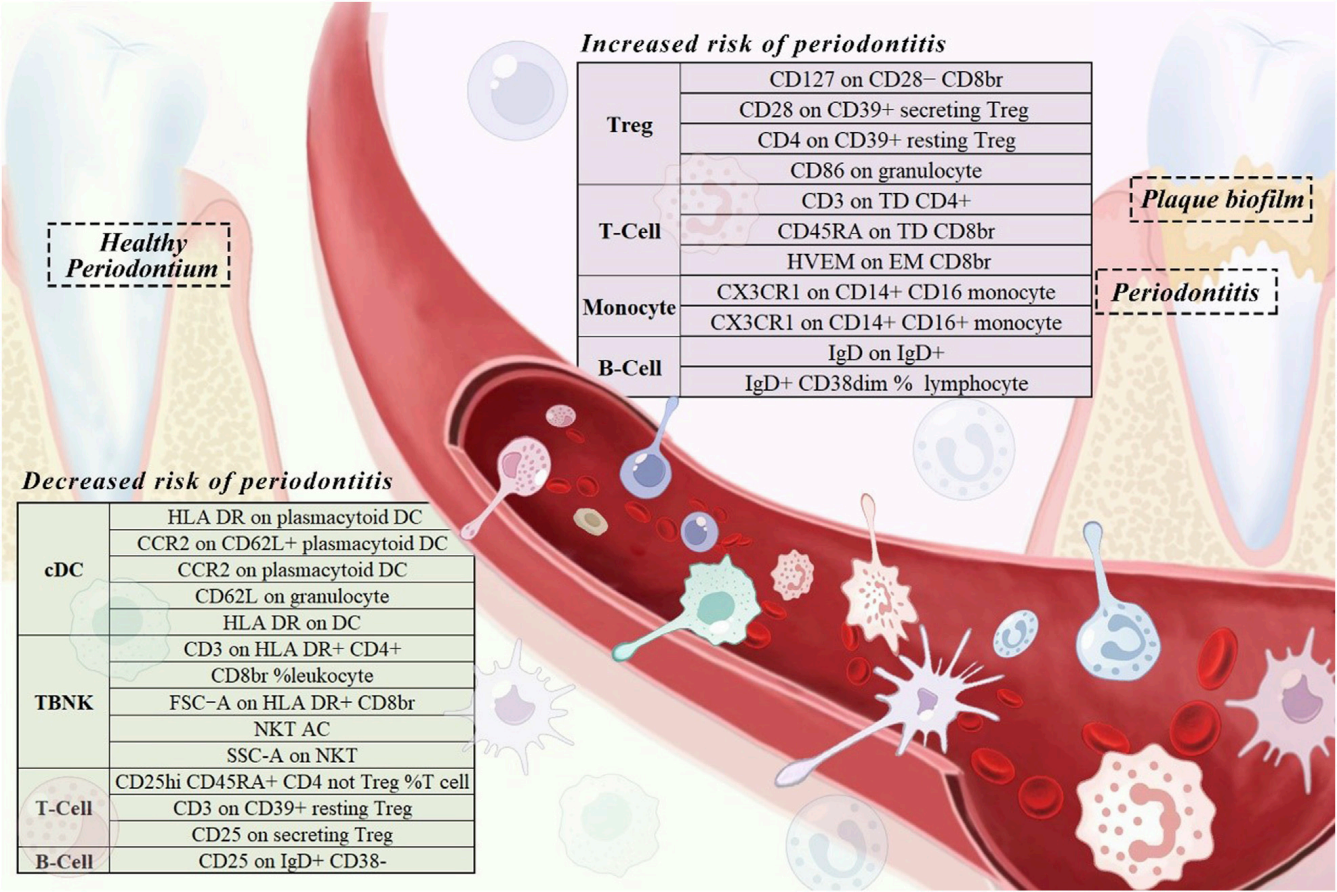
A sketch of immune cell phenotypes associated with periodontitis risk.

**FIGURE 4:**
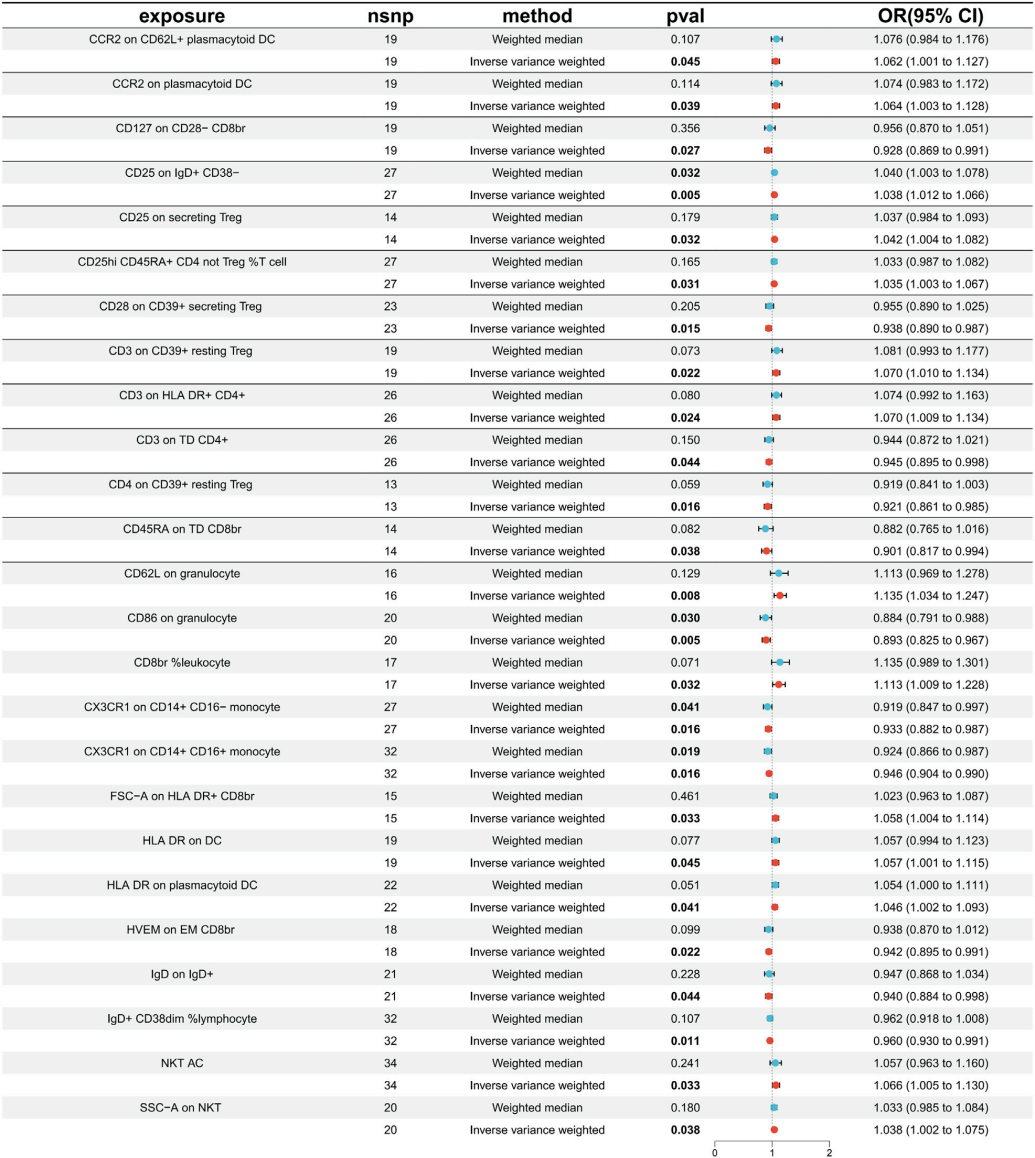
The forest plot displays causality for immune cells as an exposure factor and PD as an outcome variable. Red dots represent IVW estimates, blue dots represent Weight median estimates, and black bars indicate 95% confidence intervals for the estimates. An OR greater than one signifies an increased risk, indicating a positive association between the immune cell and the risk of PD. An OR less than one signifies a decreased risk, indicating a negative association between the immune cell and the risk of PD. If a horizontal line intersects 0, it suggests that the immune cell is not causally associated with the risk of developing PD.

**FIGURE 5:**
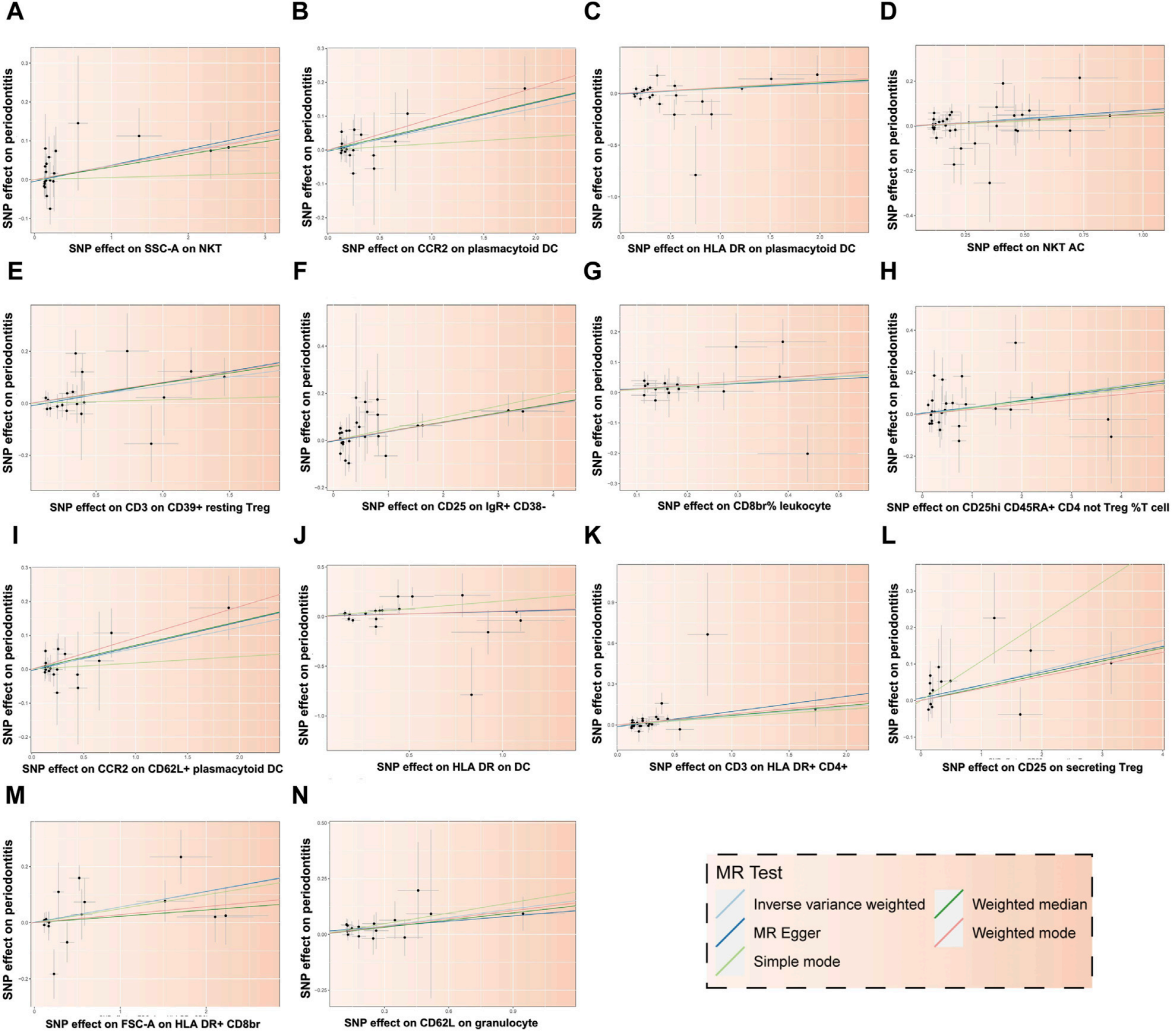
Scatter plot illustrating the relationship between 14 immune cells and the risk of PD. Each letter corresponds to a specific immune cell: **(A)** SSC-A on NKT, **(B)** CCR2 on plasmacytoid DC, **(C)** HLA DR on plasmacytoid DC, **(D)** NKT AC, **(E)** CD3 on CD39^+^ resting Treg, **(F)** CD25 on IgR+ CD38^−^, **(G)** CD8br% leukocyte, **(H)** CD25hi CD45RA + CD4 not Treg %T cell, **(I)** CCR2 on CD62L + plasmacytoid DC, **(J)** HLA DR on DC, **(K)** CD3 on HLA DR+ CD4^+^, **(L)** CD25 on secreting Treg, **(M)** FSC-A on HLA DR + CD8br, **(N)** CD62L on granulocyte. The *X*-axis represents the effect of SNPs on exposure factors, and the *Y*-axis represents the effect of SNPs on outcome factors. The positive slope of the fitted line indicates that these 14 immune cells are deleterious factors for PD, implying a negative association with the risk of PD.

The specific SNPs for each immune cell phenotype are listed in Supplementary Table S1. The results of the preliminary MR analysis of the 731 immune cell phenotypes and the risk of PD are presented in Figure 2 and Supplementary Table S2. A total of 25 immune profiles were directly and significantly associated with PD at a significance threshold of *p* < 0.05. The sketch of immune cell phenotypes associated with PD risk is shown in Figure 3. The statistical results and visualized forest plots are shown in Supplementary Table S3 and Figure 4. These include 3 cases in the B cell group, 5 cases in the cDC group, 5 cases in the mature T-cell group, 3 cases in the TBNK group, 5 cases in the monocyte group, and 7 cases in the Treg group. The results of MR analysis using a random effects model with the IVW method identified 14 immune cell characteristics as having a potential positive causal relationship with an increased risk of PD (Figure 5). These included CD127 on CD28^−^ CD8br, Treg (OR = 0.928, 95% CI = 0.869-0.991, *p* = 0.027), CD28 on CD39^+^ secreting Treg, Treg (OR = 0.938, 95% CI = 0.890 to 0.987, *p* = 0.015), CD3 on TD CD4^+^, Maturation stages of T cell (OR = 0.945, 95% CI = 0.895 to 0.998, *p* = 0.044), CD4 on CD39^+^ resting Treg, Treg (OR = 0.921, 95% CI = 0.861 to 0.985, *p* = 0.016), CD45RA on TD CD8br, Maturation stages of T cell (OR = 0.901, 95% CI = 0.817 to 0.994, *p* = 0.038), CD86 on granulocyte, Treg (OR = 0.893, 95% CI = 0.825 to 0.967, *p* = 0.005), CX3CR1 on CD14^+^ CD16^−^ monocyte, Monocyte (OR = 0.933, 95% CI = 0.882 to 0.987, *p* = 0.016), CX3CR1 on CD14^+^ CD16^+^ monocyte, Monocyte (OR = 0.946, 95% CI = 0.904 to 0.990, *p* = 0.016), HVEM on EM CD8br, Maturation stages of T cell (OR = 0.942, 95% CI = 0.895 to 0.991, *p* = 0.022), IgD on IgD+, B cell (OR = 0.940, 95% CI = 0.884 to 0.998, *p* = 0.044), IgD + CD38dim %lymphocyte, B cell (OR = 0.960, 95% CI = 0.930 to 0.991, *p* = 0.011).

**FIGURE 6:**
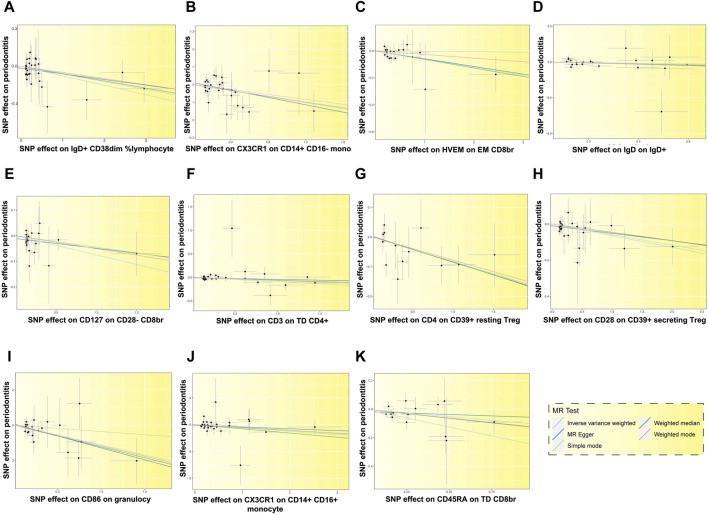
Scatter plots depicting the relationship between 11 immune cells and the risk of PD. Each letter corresponds to a specific immune cell: **(A)** IgD + CD38dim %lymphocyte, **(B)** CX3CR1 on CD14^+^ CD16-monocyte, **(C)** HVEM on EM CD8br, **(D)** IgD on IgD+, **(E)** CD127 on CD28^−^ CD8br, **(F)** CD3 on TD CD4^+^, **(G)** CD4 on CD39^+^ resting Treg, **(H)** CD28 on CD39^+^ secreting Treg, **(I)** CD86 on granulocyte, **(J)** CX3CR1 on CD14^+^ CD16^+^ monocyte, **(K)** CD45RA on TD CD8br. The slopes of the fitted lines corresponding to the relationship between the 11 immune cells and exposure to PD are less than 0, suggesting that these 11 immune cells are deleterious factors for PD, indicating a negative association with the risk of PD.

In addition, 11 types of immune cells are negatively associated with an increased risk of PD (Figure 6), including CCR2 on CD62L + plasmacytoid DC, cDC (OR = 1.062, 95% CI = 1.001 to 1.127, *p* = 0.045), CCR2 on plasmacytoid DC, cDC (OR = 1.064, 95% CI = 1.003 to 1.128, *p* = 0.039), CD25 on IgD + CD38^−^, B cell (OR = 1.038, 95% CI = 1.012 to 1.066, *p* = 0.005), CD25 on secreting Treg, Treg (OR = 1.042, 95% CI = 1.004 to 1.082, *p* = 0.032), CD25hi CD45RA + CD4 not Treg %T cell, Treg (OR = 1.035, 95% CI = 1.003 to 1.067, *p* = 0.031), CD3 on CD39^+^ resting Treg, Treg (OR = 1.070, 95% CI = 1.010 to 1.134, *p* = 0.022), CD3 on HLA DR+ CD4^+^, TBNK (OR = 1.070, 95% CI = 1.009 to 1.134, *p* = 0.024), CD62L on granulocyte, cDC (OR = 1.135, 95% CI = 1.034 to 1.247, *p* = 0.008), CD8br %leukocyte, TBNK (OR = 1.113, 95% CI = 1.009 to 1.228, *p* = 0.032), FSC−A on HLA DR + CD8br, TBNK (OR = 1.058, 95% CI = 1.004 to 1.114, *p* = 0.033), HLA DR on DC, cDC (OR = 1.057, 95% CI = 1.001 to 1.115, *p* = 0.045), HLA DR on plasmacytoid DC, cDC (OR = 1.046, 95% CI = 1.002 to 1.093, *p* = 0.041), NKT AC, TBNK (OR = 1.066, 95% CI = 1.005 to 1.130, *p* = 0.033), SSC−A on NKT, TBNK (OR = 1.038, 95% CI = 1.002 to 1.075, *p* = 0.038).

## 4 Sensitivity analysis

To verify the reliability and robustness of the results, we performed inverse MR analysis to screen for 25 immune cell phenotypes causally associated with PD, which showed that there was no significant correlation (*p* > 0.05) between the two (Supplementary Table S4). In sensitivity analysis, we found no significant heterogeneity across the selected IVs, indicating consistency in the causal estimates and supporting the validity of the IVs used in the MR analysis (Supplementary Table S5). In addition, the intercept from MR-Egger regression was not statistically significant (*p* > 0.05), suggesting no evidence of directional pleiotropy (Supplementary Table S6). MR-PRESSO analyses showed no significant outliers were detected, further supporting the robustness of the causal inferences (Supplementary Table S7). The funnel plot showed that the SNP distribution was more symmetrical, and the causality was unlikely to be affected by potential bias, indicating the stability of the results (Supplementary Figure S1 and S2). Leave-one-out analysis showed that the significant causality had no change or reversal of the combined effect after removing SNPs one by one (Supplementary Figures S3 and S4), indicating that the results were plausible. In summary, there was a consistent positive correlation between 14 immune cells and PD and a consistent negative correlation between another 11 immune cells and PD.

## 5 Discussion

The immune system plays a pivotal role in oral health, particularly in the pathogenesis of PD, and its involvement is increasingly gaining attention in both basic science and clinical research. By regulating inflammatory and immune responses, the immune system combats pathogens in the oral cavity and maintains the healthy state of oral tissues. However, the current understanding of the genetic-level relationship between the immune system and PD remains insufficient. To our knowledge, this is the first study to explore the causal relationship between immune cell phenotypes and PD. The study identified 25 immune cells correlated with the risk of PD, with further screening revealing 14 that were positively associated and 11 negatively associated with PD risk. The findings offer crucial insights for future research in this domain.

**FIGURE 7:**
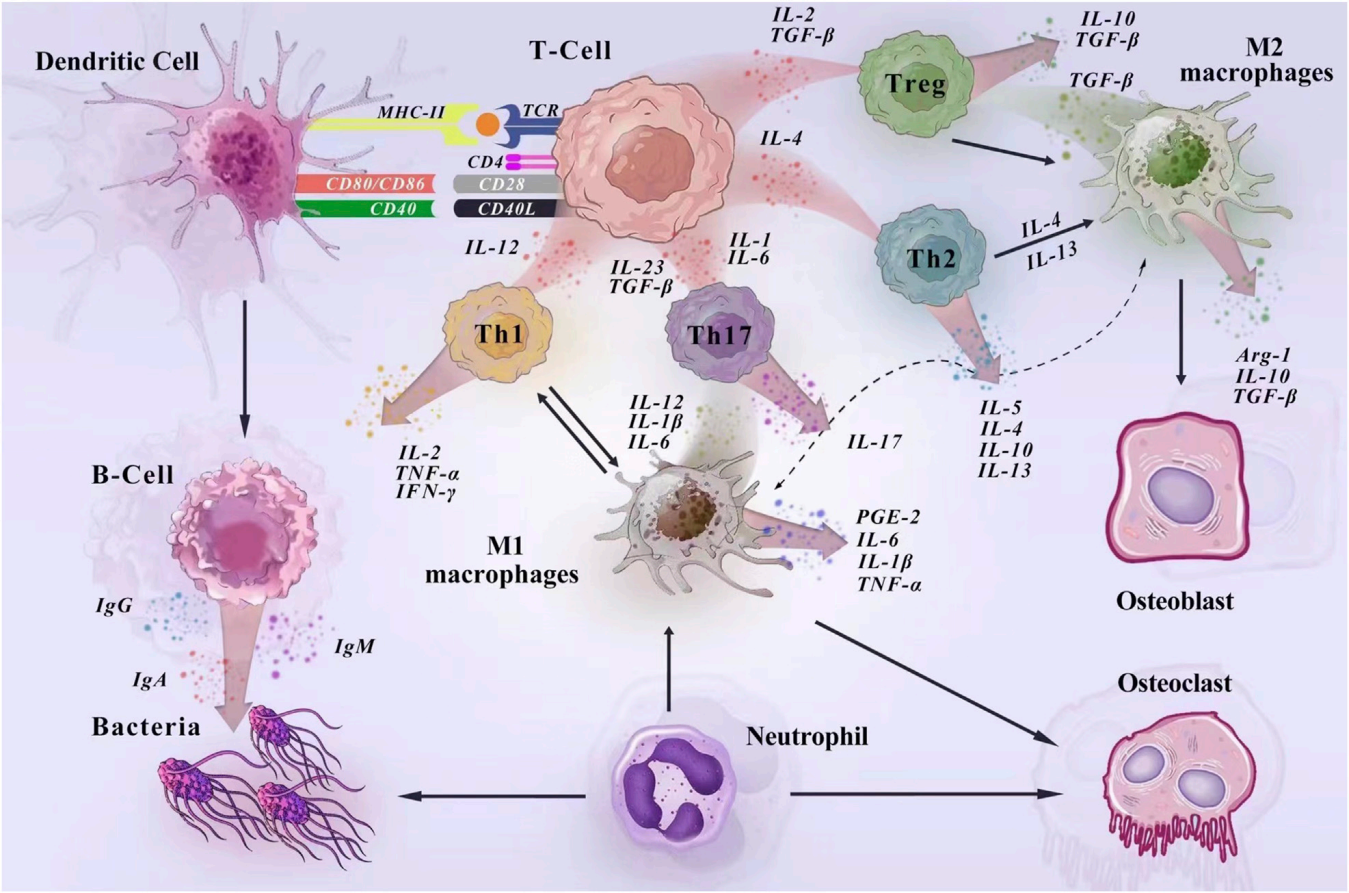
Mechanisms of immune cell regulation of PD in the host immune microenvironment. In the development of PD, dendritic cells act as antigen-presenting cells to activate T lymphocytes and regulate the acquired immune response. Surface antigens on DCs (MHC-II, CD80/86, and CD40) activate molecular signaling on T cells to stimulate naïve T cells to proliferate (El-Awady et al., 2000). Macrophages are activated in response to bacteria and their products, cytokines. In the early stage of inflammation, M1 macrophages dominate and produce pro-inflammatory factors to enhance the inflammatory response. In the late stage of inflammation, M2 macrophages release anti-inflammatory cytokines and trophic factors to play an anti-inflammatory role and promote the repair of damaged tissues. During the immune response, macrophages and T cells interact to promote macrophage activation and co-regulate inflammation (Cavalla and Hernández, 2022; Han et al., 2023). Neutrophils are an important line of defense against periodontal pathogens. They kill bacteria under the regulation of cytokines, adhesion molecules and chemokines and participate in the induction of immune response by synthesizing and releasing cytokines with immunomodulatory effects (Silva et al., 2019). During humoral immunization, when antigen-presenting cells present the information, B cells produce large amounts of antibodies to fight the bacterial (Han et al., 2023).

The mechanism by which immune cells regulate PD in the host immune microenvironment is shown in Figure 7. Dendritic cells play a crucial role in the development of PD, acting as the “conductors” of T-cell differentiation. These cells possess a remarkable ability to stimulate and regulate effector responses of T cells, thereby enhancing the inflammatory response in periodontal tissues by upregulating the activation of Th1 and Th17 cells (El-Awady et al., 2000; Meyle and Chapple, 2000). In cases of periodontal tissue infection and inflammation, Porphyromonas gingivalis can stimulate mature dendritic cells to secrete IL-12 and IFN-γ, promoting a Th1 cell response and exacerbating inflammation (Su et al., 2015; Meghil and Cutler, 2020). The results of our study indicate that dendritic cell immunophenotypes contribute to the risk of PD, including CCR2 on CD62L + plasmacytoid DC, CCR2 on plasmacytoid DC, CD62L on granulocyte, HLA DR on DC, HLA DR on plasmacytoid DC. CCR2, or C-C chemokine receptor type 2, plays a crucial role in inflammation and bone metabolic diseases. Our findings align with previous studies indicating that CCR2 has a significant inflammatory effect in periodontal tissues. Previous experiments demonstrated high expression of CCR2 in human and mouse periodontal tissues. When CCR2 was reduced, there was a decrease in inflammatory monocyte and macrophage infiltration in periodontal tissues, along with reduced levels of inflammatory mediators and osteoclasts (Jiang et al., 2022). Additionally, in periodontal tissues affected by peri-implantitis, the elimination of CCR2 resulted in reduced production of pro-inflammatory cytokines and impaired osteoclast activity, suppressing peri-implant inflammation and bone loss (Yuan et al., 2023).

T cells, also known as thymus-dependent lymphocytes, constitute the primary component of lymphocytes and serve various biological functions, including the direct killing of target cells and assisting or inhibiting antibody production by B cells (Gonzales, 2000). These cells undergo positive and negative selection processes to develop into mature T cells with immune functions, expressing either CD4^+^ or CD8^+^ (Hogquist et al., 2015). In our study, two subpopulations at different T cell maturation stages, namely, CD45RA on TD CD8br and HVEM on EM CD8br, were both associated with a reduced risk of PD. The CD8 molecule, a leukocyte differentiation antigen, is a glycoprotein present on the surface of certain T cells. It assists the T cell receptor (TCR) in recognizing antigens, participates in the transduction of T cell activation signals, and is involved in the specific killing of target cells. Previous research has demonstrated that CD8 plays diverse roles in processes associated with the inflammatory response. For instance, Nishimura et al. reported that immune and genetic depletion of CD8^+^ T cells led to reduced macrophage infiltration, adipose tissue inflammation, and improved systemic insulin resistance (Nishimura et al., 2009). In another study, Gunderson et al. found that CD8^+^ T cells induced psoriatic-like skin inflammation through IFN-γ-mediated pathways (Gunderson et al., 2013). Conversely, Wu et al. observed that CD8 T cells could regulate autoreactive Th1 and Th17 cells via IFN-γ, thereby reducing neuroretinal inflammation in mice (Wu et al., 2023). Hence, we speculate that the role of CD8 in modulating the inflammatory response may be dual, with outcomes influenced by factors such as the disease site or type, animal model, or target population.

Tregs are a subset of lymphocytes responsible for negatively regulating the body’s immune response, playing a crucial role in maintaining self-tolerance and preventing excessive damage from immune responses. During inflammation resulting from periodontal tissue infection, there is often an accumulation of Treg cells in the body (Meghil and Cutler, 2020). CD39, an extracellular nucleotide hydrolase, binds to extracellular ATP and converts it to extracellular adenosine, thereby suppressing immune responses (Muls et al., 2015). Our findings indicated that the Treg immune phenotype CD4 on CD39^+^ resting Treg was associated with a reduced risk of PD. Conversely, the CD3 on CD39^+^ resting Treg phenotype exhibited a protective effect, increasing the risk of PD. We hypothesize that these two immune phenotypes, although part of the same cellular subpopulation, may have different roles due to differences in the antigens they express (CD3 and CD4). CD3-positive T-lymphocytes represent the total population of mature T-lymphocytes in the body and can serve as specific markers for T-lymphocytes, participating in T-lymphocyte signaling processes. On the other hand, CD4-positive T lymphocytes are helper T lymphocytes, primarily enhancing phagocyte-mediated anti-infection responses and B lymphocyte-mediated humoral immune responses (Wu et al., 2022).

Mononuclear macrophages play a pivotal role in activating the host’s defense mechanisms against bacterial infections, helping maintain the delicate balance between the host and microbes (Sima et al., 2019). Bacteria and their byproducts, such as endotoxins, can activate the monocyte/macrophage system, leading to the production of numerous pro-inflammatory factors that can trigger inflammation or immune responses (Benoit et al., 2008). Our study revealed that two monocyte populations, CX3CR1 on CD14^+^CD16-monocytes and CX3CR1 on CD14^+^ CD16^+^ monocytes, were associated with a reduced risk of PD. CD14^+^ CD16-and CD14^+^ CD16^+^ are recognized as the two main subpopulations of blood monocytes, with CD14^+^ CD16^+^ monocytes being activated cells marked by pro-inflammatory cytokines, demonstrating an ability to enhance inflammatory activity and interact with endothelial cells (Ziegler-Heitbrock, 2007; Merino et al., 2011). An increased proportion of CD14^+^ CD16^+^ monocytes has been observed in both peripheral blood and gingival tissue of patients with chronic PD (Jagannathan et al., 2014). It is worth noting that our results may differ from previous studies, and we hypothesize that this discrepancy could be attributed to the consideration of other relevant cellular molecular markers, such as CX3CR1. CX3CR1, also known as Chemokine Receptor 1, acts as a receptor for CX3CL1, mediating the migration of inflammatory cells and playing a regulatory role in inflammation (Mionnet et al., 2010; Morimura et al., 2016).

B cells exhibit diverse immune functions, encompassing antibody secretion, antigen presentation, cytokine production, and the regulation of T effector cell differentiation (Cyster and Allen, 2019). Research indicates that B cells can contribute to the destruction of periodontal soft and hard tissues through the secretion of pro-inflammatory cytokines, NF-κB ligand receptor activator (RANKL), osteoclastogenic factors released by activated T cells, matrix metalloproteinases, and autoantibodies (Kawai et al., 2006; Figueredo et al., 2019). Our findings regarding 2 B cell subsets, lgD + CD38dim %ymphocyte and CD25 on IgR+ CD38^−^, showed inconsistent results. This discrepancy may be attributed to the physiological complexity of the CD38 molecule. CD38 functions as a multifaceted molecule, acting as an enzyme and playing various roles in immunoregulatory processes, including the regulation of cell recruitment, phagocytosis, antigen presentation, cytokine release, and NAD availability (Piedra-Quintero et al., 2020). Additionally, differences in other relevant markers, fluorescence characterization, and fluorescence intensity could contribute to these variations.

It is necessary to acknowledge that there are several limitations to this study. Firstly, given that this study relies on data from a European-origin population, it is important to consider the potential impact of population stratification on the observed associations. European populations are genetically diverse, and differences in allele frequencies can exist between subpopulations, such as those from different regions or with different ancestries. Considering the genetic heterogeneity among different ethnic groups, the results may vary from population to population. Therefore, whether our findings can be generalized to other ethnic groups needs to be further verified by prospective studies. In addition, GWAS provide summary statistics for the association between genetic variants and traits, rather than individual-level genotype data. While summary data offer the advantage of large sample sizes and cost-effectiveness, the limitation is the inability to directly assess and control for confounding factors or perform individual-level analyses and to capture important covariates or environmental factors that could confound genetic associations.

Furthermore, the interaction between genetic predispositions and environmental or lifestyle factors is a critical aspect of the pathogenesis of PD. While this study focuses on elucidating the genetic basis of PD through MR analyses, incorporating environmental and lifestyle factors into future analyses is essential for constructing a comprehensive risk model for the disease. By leveraging large-scale genetic datasets and comprehensive phenotypic information, future MR studies can explore gene-environment interactions and identify causal pathways underlying periodontal disease risk. Finally, variations in the definition of PD across different studies and differences in the selected GWAS datasets for PD may also impact the consistency of the conclusions.

## 6 Clinical implications and future directions

In a clinical setting, understanding the implications of immune cell phenotypes on PD is essential for developing targeted interventions and precision medicine. For instance, the identification of immune cell phenotypes associated with PD risk can facilitate patient stratification in clinical trials. Stratifying patients based on their immune cell profiles may help identify subgroups that are more likely to benefit from targeted interventions, thereby enhancing treatment efficacy and patient outcomes. Moreover, immune cell phenotypes identified in the study could serve as potential biomarkers for monitoring disease progression and treatment response in clinical trials. Incorporating these biomarkers into trial protocols can provide valuable mechanistic insights and facilitate the assessment of treatment effects on immune dysregulation in PD. Finally, the study’s findings support the development of precision medicine approaches in PD, wherein treatment strategies are tailored to individual patients based on their immune profiles. Leveraging advances in omics technologies and computational modeling can further refine patient stratification and enable personalized treatment regimens.

However, the immune response in PD is complex and involves interactions between multiple cell types and signaling pathways. Targeting specific immune cell phenotypes may not fully capture the intricacies of the immune dysregulation in PD. Therefore, translating research findings into effective clinical interventions may require a comprehensive understanding of the underlying mechanisms. 1). Individual variability: There is significant variability in immune cell phenotypes and responses among individuals, influenced by factors such as genetics, environmental exposures, and host-microbiome interactions. Developing personalized immunotherapies based on immune cell phenotypes may require tailored approaches to account for this variability. 2). Therapeutic target identification: While the study identifies immune cell phenotypes associated with PD risk, further research is needed to elucidate the specific molecular targets and pathways underlying these associations. Identifying druggable targets and developing targeted therapies may require extensive preclinical validation and translational research efforts. 3). Clinical trial design challenges: These include selecting appropriate patient populations, defining clinically relevant endpoints, and ensuring the safety and efficacy of experimental interventions. Additionally, optimizing trial designs to account for potential confounders and heterogeneity in treatment responses is essential for generating robust and generalizable results.

In conclusion, this study marks the inaugural application of two-sample MR study to unravel the causal relationship between immune cells and PD. It unveils the impact of particular immune cell phenotypes on the risk of PD, offering novel insights for targeted immunotherapeutic strategies.

## Data Availability

Publicly available datasets were analyzed in this study. This data can be found here: https://gwas.mrcieu.ac.uk. https://www.nature.com/articles/s41588-020-0684-4.

## Abbreviations

PD, Periodontitis; MR, Mendelian randomization; IV, Instrumental variable; AC, Absolute cell; MFI, Median fluorescence intensity; MP, Morphological parameters; RC, Relative cell; cDC, Conventional dendritic cell; TBNK, T-cells, B-cells, natural killer cells; Tregs, Regulatory T cells; SNPs, Single nucleotide polymorphisms; IVW, Inverse variance weighting; TCR, T cell receptor.
