# Supplementary material for: Leveraging genetics to investigate causal effects of immune cell phenotypes in periodontitis: a mendelian randomization study
Source: Front Genet. 2024 Jun 21;15:1382270. doi: 10.3389/fgene.2024.1382270 (PMC11224148; doi:10.3389/fgene.2024.1382270)
Supplement: Supplementary file 2 [file Table1.DOCX]

Supplementary Figures and Codes

## 1 Supplementary Figures


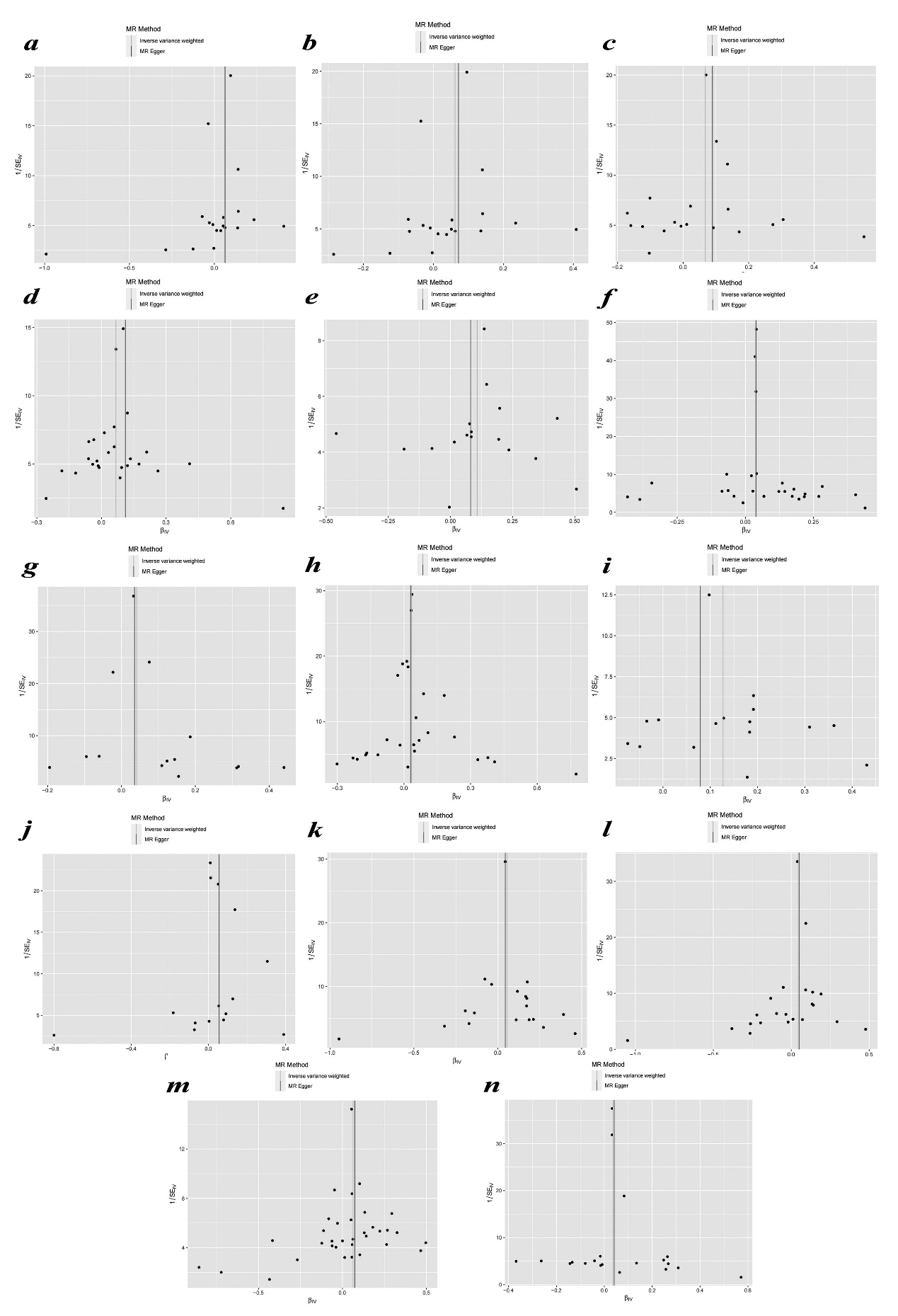


**Figure S1** Funnel plots for 14 immune cells positively associated with the risk of periodontitis. a. CCR2 on CD62L+ plasmacytoid DC. b. CCR2 on plasmacytoid DC. c. CD3 on CD39+ resting Treg. d. CD3 on HLA DR+ CD4+. e. CD8br _leukocyte. f. CD25 on IgD+ CD38-. g. CD25 on secreting Treg. h. CD25hi CD45RA+ CD4 not Treg _T cell. i. CD62L on granulocyte. j. FSC-A on HLA DR+ CD8br. k. HLA DR on DC. l. HLA DR on plasmacytoid DC. m. NKT AC. n. SSC-A on NKT.


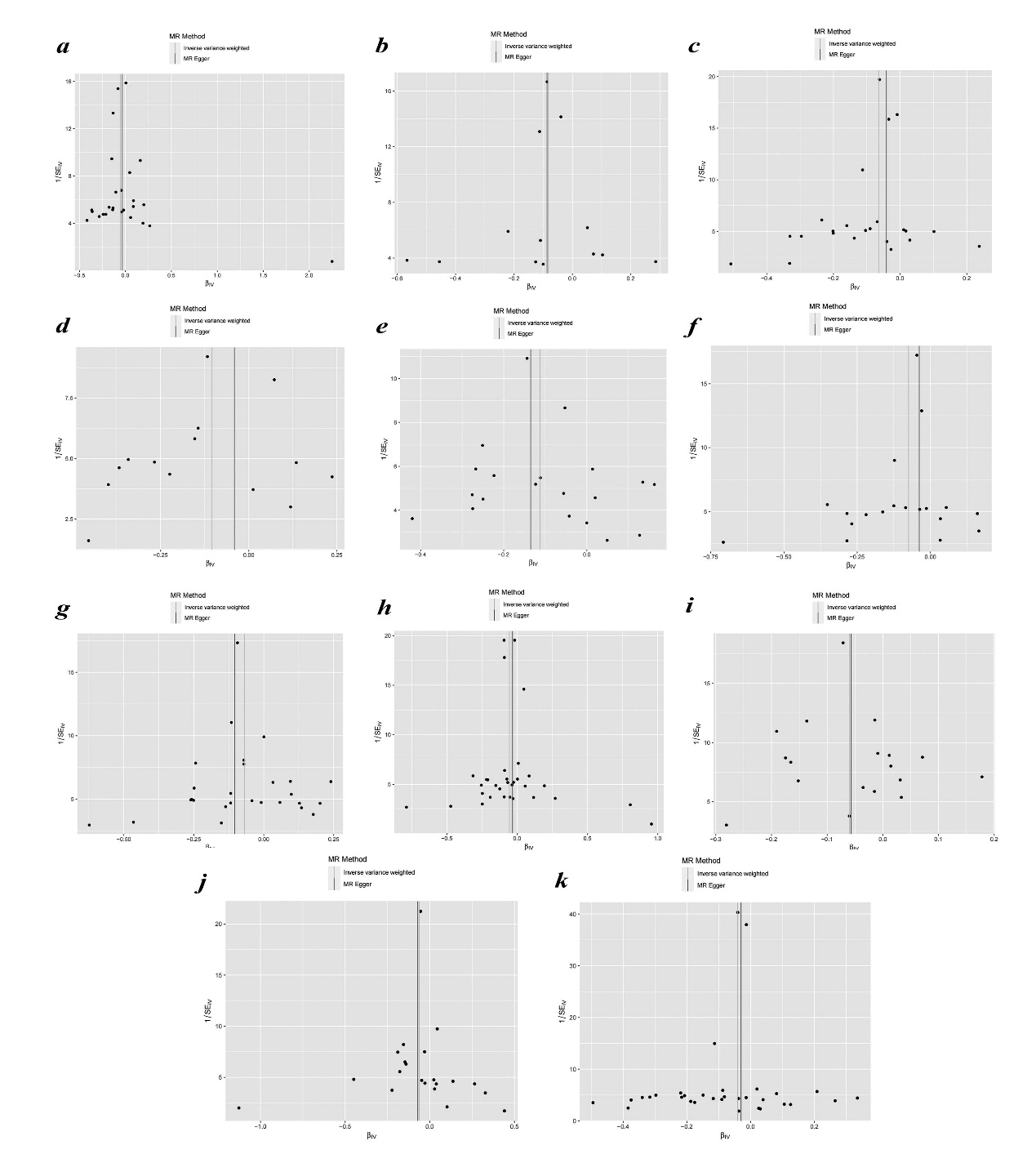


**Figure S2** Funnel plots for 11 immune cells negatively associated with the risk of periodontitis. a.CD3 on TD CD4+. b. CD4 on CD39+ resting Treg. c. CD28 on CD39+ secreting Treg. d. CD45RA on TD CD8br. e. CD86 on granulocyte. f. CD127 on CD28- CD8br. g. CX3CR1 on CD14+ CD16- monocyte. h. CX3CR1 on CD14+ CD16+ monocyte. i. HVEM on EM CD8br. j. IgD on IgD+. k. IgD+ CD38dim _lymphocyte.


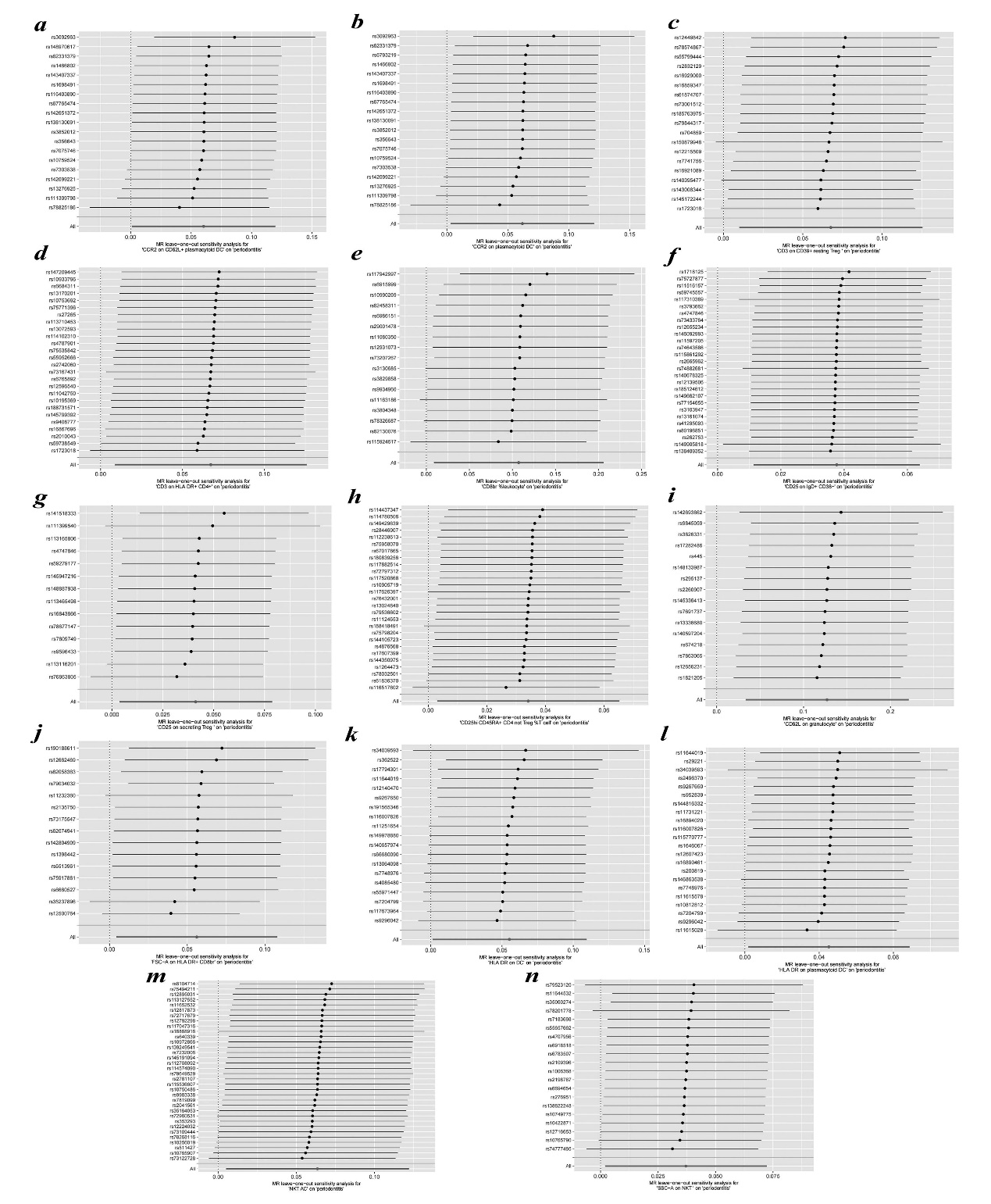
**Figure S3** Leave-one-out analyses for 14 immune cells positively associated with the risk of periodontitis.


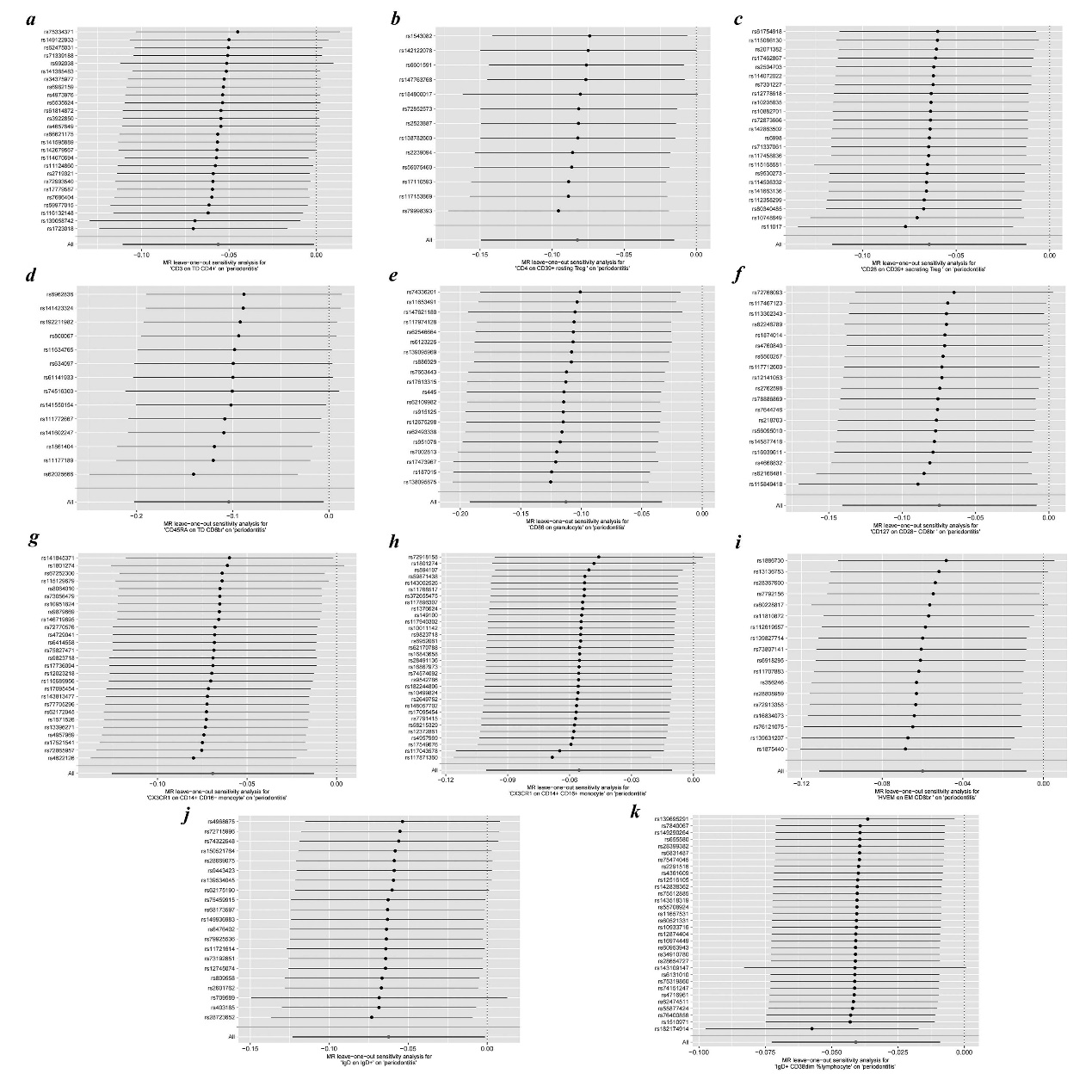
**Figure S4** Leave-one-out analyses for 11 immune cells negatively associated with the risk of periodontitis.

**2 Codes used in the MR analysis**

**2.1 MR analysis**

#install.packages("devtools")

#devtools::install_github("MRCIEU/TwoSampleMR")

library(TwoSampleMR)

#Need to convert “Raw data-Immune cell phenotype.xlsx ” to “Raw data-Immune cell phenotype.txt ” in advance

inputFile="Raw data-Immune cell phenotype.txt"

rt=read.table(inputFile, header=T, sep="\t", check.names=F)

outTab=data.frame()

for(id in rt$ID) {

expoData=extract_instruments(id,

p1 = 1e-5, p2 = 1e-5,

clump = T,

kb = 10000, r2 = 0.001)

outTab=rbind(outTab, expoData)

}

write.csv(outTab, file="exposure_data.csv", row.names=F)

#devtools::install_github("mrcieu/ieugwasr", force = TRUE)

library(ieugwasr)

inputFile="exposure_data.csv"

dat=read.csv(inputFile, header=T, sep=",", check.names=F)

dat$R2<-(2*dat$beta.exposure*dat$beta.exposure*dat$eaf.exposure*(1-dat$eaf.exposure)/(2*dat$beta.exposure*dat$beta.exposure*dat$eaf.exposure*(1-dat$eaf.exposure)+2*dat$se.exposure*dat$se.exposure*dat$samplesize.exposure*dat$eaf.exposure*(1-dat$eaf.exposure)))

dat$F<-dat$R2*(dat$samplesize.exposure-2)/(1-dat$R2)

outTab=dat[as.numeric(dat$F)>10,]

write.csv(outTab, file="exposure.F.csv", row.names=F)

#devtools::install_github("mrcieu/gwasglue",force = TRUE)

#BiocManager::install("VariantAnnotation")

#devtools::install_github("mrcieu/gwasglue", force = TRUE)

#install.packages("remotes")

#remotes::install_github("MRCIEU/TwoSampleMR")

library(VariantAnnotation)

library(gwasglue)

library(TwoSampleMR)

exposureFile="exposure.F.csv"

outcomeID="finn-b-K11_PERIODON_CHRON" outcomeName="periodontitis"

exposure_dat=read_exposure_data(filename=exposureFile,

sep = ",",

snp_col = "SNP",

beta_col = "beta.exposure",

se_col = "se.exposure",

pval_col = "pval.exposure",

effect_allele_col="effect_allele.exposure",

other_allele_col = "other_allele.exposure",

eaf_col = "eaf.exposure",

phenotype_col = "exposure",

samplesize_col = "samplesize.exposure",

chr_col="chr.exposure", pos_col = "pos.exposure",

clump=FALSE)

outcomeData=extract_outcome_data(snps=exposure_dat$SNP, outcomes=outcomeID)

write.csv(outcomeData, file="outcome.csv", row.names=F)

outcomeData$outcome=outcomeName

dat=harmonise_data(exposure_dat, outcomeData)

outTab=dat[dat$mr_keep=="TRUE",]

write.csv(outTab, file="table.SNP.csv", row.names=F)

mrResult=mr(dat)

mrTab=generate_odds_ratios(mrResult)

write.csv(mrTab, file="table.MRresult.csv", row.names=F)

heterTab=mr_heterogeneity(dat)

write.csv(heterTab, file="table.heterogeneity.csv", row.names=F)

pleioTab=mr_pleiotropy_test(dat)

write.csv(pleioTab, file="table.pleiotropy.csv", row.names=F)

pdf(file="pic.scatter_plot.pdf", width=7.5, height=7)

mr_scatter_plot(mrResult, dat)

dev.off()

res_single=mr_singlesnp(dat)

pdf(file="pic.forest.pdf", width=7, height=5.5)

mr_forest_plot(res_single)

dev.off()

pdf(file="pic.funnel_plot.pdf", width=7, height=6.5)

mr_funnel_plot(singlesnp_results = res_single)

dev.off()

pdf(file="pic.leaveoneout.pdf", width=7, height=5.5)

mr_leaveoneout_plot(leaveoneout_results = mr_leaveoneout(dat))

dev.off()

mrFile="table.MRresult.csv"

pleFile="table.pleiotropy.csv"

rt=read.csv(mrFile, header=T, sep=",", check.names=F)

ivw=data.frame()

for(immuneCell in unique(rt$exposure)){

immData=rt[rt$exposure==immuneCell,]

if(immData[immData$method=="Inverse variance weighted","pval"]<0.05){

if(sum(immData$or>1)==nrow(immData) | sum(immData$or<1)==nrow(immData)){

ivw=rbind(ivw, immData)

}

}

}

pleRT=read.csv(pleFile, header=T, sep=",", check.names=F)

pleRT=pleRT[pleRT$pval>0.05,]

immuneLists=as.vector(pleRT$exposure)

outTab=ivw[ivw$exposure %in% immuneLists,]

write.csv(outTab, file="IVW.filter.csv", row.names=F)

#install.packages("remotes")

#remotes::install_github("MRCIEU/TwoSampleMR")

exposureFile="exposure.F.csv"

outcomeFile="outcome.csv"

sigImmuneFile="IVW.filter.csv"

outcomeName="periodontitis"

rt=read.csv(exposureFile, header=T, sep=",", check.names=F)

sigImmune=read.csv(sigImmuneFile, header=T, sep=",", check.names=F)

for(immuneCell in unique(sigImmune$exposure)){

i=gsub("\\%|\\/", "_", immuneCell)

singleExposureFile=paste0(i, ".exposure.csv")

exposure_set=rt[rt$exposure==immuneCell,]

write.csv(exposure_set, file=singleExposureFile, row.names=F)

exposure_dat=read_exposure_data(filename=singleExposureFile,

sep = ",",

snp_col = "SNP",

beta_col = "beta.exposure",

se_col = "se.exposure",

pval_col = "pval.exposure",

effect_allele_col="effect_allele.exposure",

other_allele_col = "other_allele.exposure",

eaf_col = "eaf.exposure",

phenotype_col = "exposure",

samplesize_col = "samplesize.exposure",

chr_col="chr.exposure", pos_col = "pos.exposure",

clump=FALSE)

outcome_data=read_outcome_data(snps=exposure_dat$SNP,

filename="outcome.csv", sep = ",",

snp_col = "SNP",

beta_col = "beta.outcome",

se_col = "se.outcome",

effect_allele_col = "effect_allele.outcome",

other_allele_col = "other_allele.outcome",

pval_col = "pval.outcome",

eaf_col = "eaf.outcome")

outcome_data$outcome=outcomeName

dat=harmonise_data(exposure_dat, outcome_data)

outTab=dat[dat$mr_keep=="TRUE",]

write.csv(outTab, file=paste0(i, ".table.SNP.csv"), row.names=F)

presso=run_mr_presso(dat)

write.csv(presso[[1]]$`MR-PRESSO results`$`Global Test`, file=paste0(i, ".table.MR-PRESSO_Global.csv"))

write.csv(presso[[1]]$`MR-PRESSO results`$`Outlier Test`, file=paste0(i, ".table.MR-PRESSO_Outlier.csv"))

mrResult=mr(dat)

mrTab=generate_odds_ratios(mrResult)

write.csv(mrTab, file=paste0(i, ".table.MRresult.csv"), row.names=F)

heterTab=mr_heterogeneity(dat)

write.csv(heterTab, file=paste0(i, ".table.heterogeneity.csv"), row.names=F)

pleioTab=mr_pleiotropy_test(dat)

write.csv(pleioTab, file=paste0(i, ".table.pleiotropy.csv"), row.names=F)

**Scatter plot (Figures 5 and 6)**

pdf(file=paste0(i, ".scatter_plot.pdf"), width=7, height=6.5)

p1=mr_scatter_plot(mrResult, dat)

print(p1)

dev.off()

res_single=mr_singlesnp(dat)

pdf(file=paste0(i, ".forest.pdf"), width=6.5, height=5)

p2=mr_forest_plot(res_single)

print(p2)

dev.off()

**Funnel_plot (Figures S1 and S2)**

pdf(file=paste0(i, ".funnel_plot.pdf"), width=6.5, height=6)

p3=mr_funnel_plot(singlesnp_results = res_single)

print(p3)

dev.off()

**Leaveoneout result (Figures S3 and S4)**

pdf(file=paste0(i, ".leaveoneout.pdf"), width=6.5, height=5)

p4=mr_leaveoneout_plot(leaveoneout_results = mr_leaveoneout(dat))

print(p4)

dev.off()

}

**Forest plot (Figure 4)**

#install.packages("grid")

#install.packages("readr")

#install.packages("forestploter")

library(grid)

library(readr)

library(forestploter)

selectMethod=c("Inverse variance weighted", "Weighted median") #Choose the method of presentation

setwd("D:\\Forest plot") #Sett up the working directory

files=dir() #Get all files in the directory

files=grep("csv$", files, value=T) #Extract files ending in csv

#Read the results of MR analysis

data=data.frame()

for(i in files){

rt=read.csv(i, header=T, sep=",", check.names=F)

data=rbind(data, rt)

}

data=data[(data$method %in% selectMethod),]

lineVec=cumsum(c(1,table(data[,"exposure"])))

#Organize the data

data$' ' <- paste(rep(" ", 10), collapse = " ")

data$'OR(95% CI)'=ifelse(is.na(data$or), "", sprintf("%.3f (%.3f to %.3f)", data$or, data$or_lci95, data$or_uci95))

data$pval = ifelse(data$pval<0.001, "<0.001", sprintf("%.3f", data$pval))

data$exposure = ifelse(is.na(data$exposure), "", data$exposure)

data$nsnp = ifelse(is.na(data$nsnp), "", data$nsnp)

data[duplicated(data$exposure),]$exposure=""

#Prepare the graphic parameters

tm <- forest_theme(base_size = 20, #Size of graphics

#Confidence interval shape, line type, width, color, height of vertical lines at both ends

ci_pch = 16, ci_lty = 1, ci_lwd = 1.5, ci_col = "black", ci_Theight = 0.2,

#Reference line shape, width, and color

refline_lty="dashed", refline_lwd=1, refline_col="grey20",

#Size of the x-axis scale font

xaxis_cex=1,

#Footnote size, color

footnote_cex = 0.6, footnote_col = "blue")

#Plot

plot <- forestploter::forest(data[, c("exposure","nsnp","method","pval"," ","OR(95% CI)")],

est = data$or,

lower = data$or_lci95,

upper = data$or_uci95,

ci_column = 5, #Columns where credible intervals are located

ref_line = 1, #Position of the reference line

xlim = c(0.75, 1.25), #Range of X-axis

theme = tm, #Parameters of the graph

)

#Modify the color of the plausible intervals in the graph

boxcolor = c("#E64B35","#4DBBD5","#00A087","#3C5488","#F39B7F","#8491B4","#91D1C2","#DC0000","#7E6148")

boxcolor = boxcolor[as.numeric(as.factor(data$method))]

for(i in 1:nrow(data)){

plot <- edit_plot(plot, col=5,row = i, which = "ci", gp = gpar(fill = boxcolor[i],fontsize=25)) # Change the columns of col, box

}

#Set the font for pvalue

pos_bold_pval = which(as.numeric(gsub('<',"",data$pval))<0.05)

if(length(pos_bold_pval)>0){

for(i in pos_bold_pval){

plot <- edit_plot(plot, col=4,row = i, which = "text", gp = gpar(fontface="bold")) # Change col pvalue column

}

}

#Add a line segment to the graph

plot <- add_border(plot, part = "header", row =1,where = "top",gp = gpar(lwd =2))

plot <- add_border(plot, part = "header", row = c(1,3,5,7,9,11,13,15,17,19,21,23,25), gp = gpar(lwd =1))

#Set the font size and center the text

plot <- edit_plot(plot, col=1:ncol(data),row = 1:nrow(data), which = "text", gp = gpar(fontsize=12))

plot <- edit_plot(plot, col = 1:ncol(data), which = "text",hjust = unit(0.5, "npc"),part="header",

x = unit(0.5, "npc"))

plot <- edit_plot(plot, col = 1:ncol(data), which = "text",hjust = unit(0.5, "npc"),

x = unit(0.5, "npc"))

#Output Graphics

pdf("forest.pdf", width=18, heigh=18)

print(plot)

dev.off()

**2.2 Circle plot (Figure 2)**

library(circlize)

library(ComplexHeatmap)

setwd("D:\\Circle plot")#Set working directory

circos.clear()#This command needs to be executed before re-mapping

# Need to convert “data-circle plot.xlsx” to “data-circle plot.txt” in advance

dt <- read.table("data-circle plot.txt",header=T,sep = "\t")

dt1<-dt[,c(3,4)]

dt1 <- as.matrix(dt1)

row.names(dt1)=dt[,1]

dt11=dt1[,1]

dt11 <- as.matrix(dt11)

row.names(dt11)=dt[,1]

mycol1 <- colorRamp2(c(0, 0.05, 1),c("#b9568b", "#ec7576", "#f3ceac"))#Color can be changed by yourself

ann_row = dt[,2]

ann_row <- as.matrix(ann_row)#In the circlize function, matrix is required

unique(ann_row)

###############

pdf("cir.pdf",width = 60,height = 60)

circos.par(gap.after=c(3,3,3,3,3,3,30)) #circos.par()Adjust the distance between the head and tail of the ring, the larger the value, the wider the distance#To make one of the mouths of the split larger, you can add line information

circos.heatmap(dt11,col=mycol1,

#dend.side="inside",#dend.side：Controls the direction of the row clustering tree, inside for displaying in the inner circle, outside for displaying in the outer circle.

rownames.side="outside",#rownames.side：Controls the direction of the matrix row names, same as dend.side; but note that they cannot be on the same side, must be one inside and one outside.

track.height = 0.04, #Height of the track, the larger the value the thicker the ring is

rownames.col="black",

bg.border="black", #Background edge color

split = ann_row,#Split heatmaps with line annotations

show.sector.labels = F,

rownames.cex=1,#font size

rownames.font=1,#Thickness of font

cluster=FALSE

)

row_mean <- as.numeric(dt1[, 2])

circos.track(

ylim = range(row_mean), panel.fun = function(x, y) {

# Get the row average of the data corresponding to the current sector

y = row_mean[CELL_META$subset]

# Arrange row means in clustering order

y = y[CELL_META$row_order]

# Add lines and points

circos.lines(CELL_META$cell.xlim, c(1,1), lty = 2, col = "grey50",border = "blue")

circos.points(seq_along(y) -1, y, col = ifelse(y > 1, "#3b5391", "#a54554"),cex = 1,

pch = 16

)

},

cell.padding = c(0.01, 0, 0.01, 0),track.height = 0.04

)

#

dt2<-dt[,c(5,6)]

dt21=dt2[,1]

mycol2 <- colorRamp2(c(0, 0.05, 1),c("#3b5284", "#5ba8a0", "#cbe54e"))

circos.heatmap(dt21,col=mycol2,

#dend.side="inside",#dend.side：Control the direction of the row clustering tree, inside for displaying in the inner circle, outside for displaying in the outer circle

rownames.side="outside",#rownames.side：Controls the direction of the matrix row names, same as dend.side; but note that they cannot be on the same side, must be one inside and one outside

track.height = 0.04, #Height of the track, the larger the value the thicker the ring is

rownames.col="black",

bg.border="black", #Background edge color

split = ann_row,#Split heatmaps with line annotations

show.sector.labels = F,

rownames.cex=0.8,#font size

rownames.font=1,#Thickness of font

cluster=FALSE

)

row_mean <- dt2[, 2]

circos.track(

ylim = range(row_mean), panel.fun = function(x, y) {

# Get the row average of the data corresponding to the current sector

y = row_mean[CELL_META$subset]

# Arrange row means in clustering order

y = y[CELL_META$row_order]

# Add lines and points

circos.lines(CELL_META$cell.xlim, c(1,1), lty = 2, col = "grey50")

circos.points(seq_along(y) - 1, y, pch=16,col = ifelse(y > 1, "#3b5391", "#a54554"),cex = 1

)

},

cell.padding = c(0.01, 0, 0.01, 0),track.height = 0.04

)

dt3<-dt[,c(7,8)]

dt31=dt3[,1]

mycol3 <- colorRamp2(c(0, 0.05, 1),c("#d46c4e", "#f9ad6a", "#f9e07f"))

circos.heatmap(dt31,col=mycol3,

#dend.side="inside",#dend.side：Control the direction of the row clustering tree, inside for displaying in the inner circle, outside for displaying in the outer circle

rownames.side="outside",#rownames.side：Controls the direction of the matrix row names, same as dend.side; but note that they cannot be on the same side, must be one inside and one outside

track.height = 0.04, #Height of the track, the larger the value the thicker the ring is

rownames.col="black",

bg.border="black", #Background edge color

split = ann_row,#Split heatmaps with line annotations

show.sector.labels = F,

rownames.cex=0.8,#Font size

rownames.font=1,#Thickness of font

cluster=FALSE

)

row_mean <- dt3[, 2]

circos.track(

ylim = range(row_mean), panel.fun = function(x, y) {

# Get the row average of the data corresponding to the current sector

y = row_mean[CELL_META$subset]

# Arrange row means in clustering order

y = y[CELL_META$row_order]

# Add lines and points

circos.lines(CELL_META$cell.xlim, c(1,1), lty = 2, col = "grey50")

circos.points(seq_along(y) - 1, y, pch=16,col = ifelse(y > 1, "#3b5391", "#a54554"),cex = 1

)

},

cell.padding = c(0.01, 0, 0.01, 0),track.height = 0.04

)

dt4<-dt[,c(9,10)]

dt41=dt4[,1]

mycol4 <- colorRamp2(c(0, 0.05, 1),c("#015c92", "#2d82b5", "#bce6ff"))

circos.heatmap(dt41,col=mycol4,

#dend.side="inside",#dend.side：Control the direction of the row clustering tree, inside for displaying in the inner circle, outside for displaying in the outer circle

rownames.side="outside",#rownames.side：Control the direction of the matrix row names, same as dend.side; but note that they cannot be on the same side, must be one inside and one outside

track.height = 0.04, #Height of the track, the larger the value the thicker the ring is

rownames.col="black",

bg.border="black", #Background edge color

split = ann_row,#Split heatmaps with line annotations

show.sector.labels = F,

rownames.cex=0.8,#Font size

rownames.font=1,#Thickness of font

cluster=FALSE

)

row_mean <- dt4[, 2]

circos.track(

ylim = range(row_mean), panel.fun = function(x, y) {

#Get the row average of the data corresponding to the current sector

y = row_mean[CELL_META$subset]

# Arrange row means in clustering order

y = y[CELL_META$row_order]

# Add lines and points

circos.lines(CELL_META$cell.xlim, c(1,1), lty = 2, col = "grey50")

circos.points(seq_along(y) - 1, y, pch=16,col = ifelse(y > 1, "#3b5391", "#a54554"),cex = 1

)

},

cell.padding = c(0.01, 0, 0.01, 0),track.height = 0.04

)

dt5<-dt[,c(11,12)]

dt51=dt5[,1]

mycol5 <- colorRamp2(c(0, 0.05, 1),c("#e01516", "white", "#20acbd"))

circos.heatmap(dt51,col=mycol5,

#dend.side="inside",#dend.side：Control the direction of the row clustering tree, inside for displaying in the inner circle, outside for displaying in the outer circle

rownames.side="outside",#rownames.side：Control the direction of the matrix row names, same as dend.side; but note that they cannot be on the same side, must be one inside and one outside

track.height = 0.04, #Height of the track, the larger the value the thicker the ring is

rownames.col="black",

bg.border="black", #Background edge color

split = ann_row,#Split heatmaps with line annotations

show.sector.labels = F,

rownames.cex=0.8,#Font size

rownames.font=1,#Thickness of font

cluster=FALSE

)

row_mean <- dt5[, 2]

circos.track(

ylim = range(row_mean), panel.fun = function(x, y) {

# Get the row average of the data corresponding to the current sector

y = row_mean[CELL_META$subset]

# Arrange row means in clustering order

y = y[CELL_META$row_order]

# Add lines and points

circos.lines(CELL_META$cell.xlim, c(1,1), lty = 2, col = "grey50")

circos.points(seq_along(y) - 1, y, pch=16,col = ifelse(y > 1, "#3b5391", "#a54554"),cex = 1

)

},

cell.padding = c(0.01, 0, 0.01, 0),track.height = 0.04

)

name<-c("IVW_P","IVW_OR","MR-EGGER_P","MR-EGGER_OR","Simple mode_P","Simple mode_OR",

"Weight Median_P","Weight Median_OR",

"Weight Mode_P","Weight Mode_OR")

#name<-c("IVW_P","IVW_OR","MR-EGGER_P","MR-EGGER_OR","Weight Median_P","Weight Median_OR"

# )

circos.track(track.index=get.current.track.index(),panel.fun=function(x,y){

if(CELL_META$sector.numeric.index==1){ #if(CELL_META$sector.numeric.index == 3) { # the last sector

cn=rev(name)

n=length(cn)

circos.text(rep(CELL_META$cell.xlim[2],n)+convert_x(-380,"mm"),#x-coordinate

1.25*(0.1:n+0.7),#Adjust the y-coordinate

cn,cex=2.7,adj=c(0,1),facing="bending.inside")}

},bg.border=NA)

circos.trackPlotRegion(ylim = c(0,0.1),track.height = 0.01,bg.border = NA,

panel.fun = function(x, y) {

chr = CELL_META$sector.index

xlim = CELL_META$xlim

ylim = CELL_META$ylim

circos.text(mean(xlim),mean(ylim),chr,cex = 2.5,

col = "black",facing = "bending.inside", niceFacing = F)

})

dev.off()

circos.clear()#

pdf("1.pdf",width = 5,height = 5)

lg=Legend(title="IVW_Pval",

title_gp = gpar(fontsize = 10),

col_fun=mycol1,direction = c("horizontal"),grid_height = unit(5, "mm"),

grid_width = unit(10, "mm"),legend_gp = gpar(fontsize = 50))

grid.draw(lg)

dev.off()

pdf("2.pdf",width = 5,height = 5)

lg=Legend(title="MR-EGGER_Pval",

title_gp = gpar(fontsize = 10),

col_fun=mycol2,direction = c("horizontal"),grid_height = unit(5, "mm"),

grid_width = unit(10, "mm"),legend_gp = gpar(fontsize = 50))

grid.draw(lg)

dev.off()

pdf("3.pdf",width = 5,height = 5)

lg=Legend(title="Simple mode_Pval",

title_gp = gpar(fontsize = 10),

col_fun=mycol3,direction = c("horizontal"),grid_height = unit(5, "mm"),

grid_width = unit(10, "mm"),legend_gp = gpar(fontsize = 50))

grid.draw(lg)

dev.off()

pdf("4.pdf",width = 5,height = 5)

lg=Legend(title="Weight Median_Pval",

title_gp = gpar(fontsize = 10),

col_fun=mycol4,direction = c("horizontal"),grid_height = unit(5, "mm"),

grid_width = unit(10, "mm"),legend_gp = gpar(fontsize = 50))

grid.draw(lg)

dev.off()

pdf("5.pdf",width = 5,height = 5)

lg=Legend(title="Weight Mode_Pval",

title_gp = gpar(fontsize = 10),

col_fun=mycol5,direction = c("horizontal"),grid_height = unit(5, "mm"),

grid_width = unit(10, "mm"),legend_gp = gpar(fontsize = 50))

grid.draw(lg)

dev.off()
