# Supplementary material for: Leveraging genetics to investigate causal effects of immune cell phenotypes in periodontitis: a mendelian randomization study
Source: Front Genet. 2024 Jun 21;15:1382270. doi: 10.3389/fgene.2024.1382270 (PMC11224148; doi:10.3389/fgene.2024.1382270)
Supplement: Supplementary file 3 [file Table3.DOCX]

**STROBE-MR checklist of recommended items to address in reports of Mendelian randomization studies**^1^ ^2^

| **Item No.** | **Section** | **Checklist item** | Page No. | Relevant text from manuscript |
| --- | --- | --- | --- | --- |
| 1 | **TITLE and ABSTRACT** | Indicate Mendelian randomization (MR) as the study’s design in the title and/or the abstract if that is a main purpose of the study | 1-2 | Leveraging Genetics to Investigate Causal Effects of Immune Cell Phenotypes in Periodontitis: A Mendelian Randomization Study |
|  | **INTRODUCTION** |  |  |  |
| 2 | **Background** | Explain the scientific background and rationale for the reported study. What is the exposure? Is a potential causal relationship between exposure and outcome plausible? Justify why MR is a helpful method to address the study question | 2-3 | Immune cells are dynamic in the inflammatory environment and play a key role in eradicating periodontal pathogens, modulating immune responses, and instigating tissue destruction. Identifying specific immune cell phenotypes associated with periodontitis risk is essential for targeted immunotherapeutic interventions. However, the role of certain specific immune cell phenotypes in the development of periodontitis is unknown. Mendelian randomization offers a novel approach to reveal causality and address potential confounding factors through genetic instruments |
| 3 | **Objectives** | State specific objectives clearly, including pre-specified causal hypotheses (if any). State that MR is a method that, under specific assumptions, intends to estimate causal effects | 3 | By utilizing MR, this study aims to elucidate the causal links between 731 immune cell phenotypes and PD. This approach represents a paradigm shift in periodontal research, offering a robust framework to discern causal associations and identify potential therapeutic targets. |
|  | **METHODS** |  |  |  |
| 4 | **Study design and data sources** | Present key elements of the study design early in the article. Consider including a table listing sources of data for all phases of the study. For each data source contributing to the analysis, describe the following: |  |  |
|  | a) | Setting: Describe the study design and the underlying population, if possible. Describe the setting, locations, and relevant dates, including periods of recruitment, exposure, follow-up, and data collection, when available. | 3 | The schematic diagram of the MR study investigating the causal effects of immune cells phenotypes on PD is shown in Figure 1. |
|  | b) | Participants: Give the eligibility criteria, and the sources and methods of selection of participants. Report the sample size, and whether any power or sample size calculations were carried out prior to the main analysis | 3 | The study encompassed 3,046 European adults diagnosed with PD and a control sample of 195,395 European adults, retrieved from GWAS data. |
|  | c) | Describe measurement, quality control and selection of genetic variants | 3 | The dataset for periodontal disease (PD) in this study was obtained from the OpenGWAS database, focusing on European samples (https://gwas.mrcieu.ac.uk). |
|  | d) | For each exposure, outcome, and other relevant variables, describe methods of assessment and diagnostic criteria for diseases | 3 | Summary statistics for 731 immune traits were obtained from the GWAS catalog database, accessible through accession numbers GCST09001391 to GCST90002121. These immunophenotypes covered various parameters, including absolute cell (AC) counts (n=118), median fluorescence intensity (MFI) reflecting surface antigen levels (n=389), morphological parameters (MP) (n=32), and relative cell (RC) counts (n=192). Specifically, MFI, AC, and RC features included panels such as B-cells, conventional dendritic cells (cDCs), T-cell maturation stages, monocytes, myeloid cells, TBNK (T-cells, B-cells, natural killer cells), and regulatory T cells (Tregs). The MP feature included cDC and TBNK panels. |
|  | e) | Provide details of ethics committee approval and participant informed consent, if relevant |  |  |
| 5 | **Assumptions** | Explicitly state the three core IV assumptions for the main analysis (relevance, independence and exclusion restriction) as well assumptions for any additional or sensitivity analysis | 3 | Selection of IVs adhered to three hypotheses: (1) the correlation hypothesis, indicating a strong correlation between IVs and immune cell traits; (2) the exclusivity hypothesis, affirming no direct relationship between IVs and PD traits; and (3) the independence hypothesis, ensuring no correlation between IVs and confounding factors |
| 6 | **Statistical methods: main analysis** | Describe statistical methods and statistics used |  |  |
|  | a) | Describe how quantitative variables were handled in the analyses (i.e., scale, units, model) | 3 | In the MR analysis, single nucleotide polymorphisms (SNPs) served as instrumental variables (IVs) to assess the causal relationship between immune cell traits and PD. |
|  | b) | Describe how genetic variants were handled in the analyses and, if applicable, how their weights were selected | 3 | IVs, chosen based on genome-wide significance (p =1×10-5) and conditions of chain imbalance threshold (r2=0.001, kb=10000), underwent further refinement. To exclude the effect of bias of weak IVs, the F statistic of each IV was calculated, with IVs having F < 10 being excluded, and only those with F > 10 included for MR analysis. In this study, a reverse MR analysis of PD and immune cells was performed to screen eligible IVs at a threshold of p = 5 × 10-6. |
|  | c) | Describe the MR estimator (e.g. two-stage least squares, Wald ratio) and related statistics. Detail the included covariates and, in case of two-sample MR, whether the same covariate set was used for adjustment in the two samples | 3-4 |  |
|  | d) | Explain how missing data were addressed | 3-4 |  |
|  | e) | If applicable, indicate how multiple testing was addressed | 3-4 |  |
| 7 | **Assessment of assumptions** | Describe any methods or prior knowledge used to assess the assumptions or justify their validity | 4 | The evaluation of the causal relationship between immune cells and PD was primarily conducted using R software (version 4.3.2), MRPRESSO (1.0), and Two Sample MR (0.5.8). MR analysis utilized inverse variance weighting, weighted median and MR-Egger methods. |
| 8 | **Sensitivity analyses and additional analyses** | Describe any sensitivity analyses or additional analyses performed (e.g. comparison of effect estimates from different approaches, independent replication, bias analytic techniques, validation of instruments, simulations) | 4 | Methodological robustness was ensured through Cochran's Q test, MR-Egger regression, MR-PRESSO, and Leave-One-Out analysis. |
| 9 | **Software and pre-registration** |  |  |  |
|  | a) | Name statistical software and package(s), including version and settings used | 4 | The evaluation of the causal relationship between immune cells and PD was primarily conducted using R software (version 4.3.2), MRPRESSO (1.0), and Two Sample MR (0.5.8). |
|  | b) | State whether the study protocol and details were pre-registered (as well as when and where) |  | NOT |
|  | **RESULTS** |  |  |  |
| 10 | **Descriptive data** |  |  |  |
|  | a) | Report the numbers of individuals at each stage of included studies and reasons for exclusion. Consider use of a flow diagram | 4 | The specific SNPs for each immune cell phenotype are listed in Table S1. The results of the preliminary MR analysis of the 731 immune cell phenotypes and the risk of PD are presented in Figure 2 and Table S2. |
|  | b) | Report summary statistics for phenotypic exposure(s), outcome(s), and other relevant variables (e.g. means, SDs, proportions) | 4 | The specific SNPs for each immune cell phenotype are listed in Table S1. |
|  | c) | If the data sources include meta-analyses of previous studies, provide the assessments of heterogeneity across these studies |  | NOT |
|  | d) | For two-sample MR:  i.  Provide justification of the similarity of the genetic variant-exposure associations between the exposure and outcome samples  ii.  Provide information on the number of individuals who overlap between the exposure and outcome studies | 4 | A total of 559 SNPS were included in the two-sample MR analysis based on the SNPS screening criteria. |
| 11 | **Main results** |  |  |  |
|  | a) | Report the associations between genetic variant and exposure, and between genetic variant and outcome, preferably on an interpretable scale | 4 | The specific SNPs for each immune cell phenotype are listed in Tables S1 and S2. |
|  | b) | Report MR estimates of the relationship between exposure and outcome, and the measures of uncertainty from the MR analysis, on an interpretable scale, such as odds ratio or relative risk per SD difference | 4 | The estimation of the results is shown in Part 3.2 |
|  | c) | If relevant, consider translating estimates of relative risk into absolute risk for a meaningful time period | 4 |  |
|  | d) | Consider plots to visualize results (e.g. forest plot, scatterplot of associations between genetic variants and outcome versus between genetic variants and exposure) | 5 | The plots to visualize results is shown in Figures 4-6. |
| 12 | **Assessment of assumptions** |  |  |  |
|  | a) | Report the assessment of the validity of the assumptions | 5 |  |
|  | b) | Report any additional statistics (e.g., assessments of heterogeneity across genetic variants, such as *I^2^*, Q statistic or E-value) | 5-6 |  |
| 13 | **Sensitivity analyses and additional analyses** |  |  |  |
|  | a) | Report any sensitivity analyses to assess the robustness of the main results to violations of the assumptions | 6 | In sensitivity analysis, we found no significant heterogeneity across the selected IVs, indicating consistency in the causal estimates and supporting the validity of the IVs used in the MR analysis (Table S5). |
|  | b) | Report results from other sensitivity analyses or additional analyses | 6 | MR-PRESSO analyses showed no significant outliers were detected, further supporting the robustness of the causal inferences (Table S7). |
|  | c) | Report any assessment of direction of causal relationship (e.g., bidirectional MR) | 6 | To verify the reliability and robustness of the results, we performed inverse MR analysis to screen for 25 immune cell phenotypes causally associated with PD, which showed that there was no significant correlation (p > 0.05) between the two (Table S4). |
|  | d) | When relevant, report and compare with estimates from non-MR analyses | 6 |  |
|  | e) | Consider additional plots to visualize results (e.g., leave-one-out analyses) | 6 | Leave-one-out analysis showed that the significant causality had no change or reversal of the combined effect after removing SNPs one by one (Figure S3 and S4), indicating that the results were plausible. |
|  | **DISCUSSION** |  |  |  |
| 14 | **Key results** | Summarize key results with reference to study objectives | 6-9 | This is the first study to explore the causal relationship between immune cell phenotypes and PD. The study identified 25 immune cells correlated with the risk of PD, with further screening revealing 11 that were positively associated and 14 negatively associated with PD risk. The findings offer crucial insights for future research in this domain. |
| 15 | **Limitations** | Discuss limitations of the study, taking into account the validity of the IV assumptions, other sources of potential bias, and imprecision. Discuss both direction and magnitude of any potential bias and any efforts to address them | 8-9 | It is necessary to acknowledge that there are several limitations to this study. Firstly, given that this study relies on data from a European-origin population, it's important to consider the potential impact of population stratification on the observed associations. |
| 16 | **Interpretation** |  |  |  |
|  | a) | Meaning: Give a cautious overall interpretation of results in the context of their limitations and in comparison with other studies | 6-9 | While summary data offer the advantage of large sample sizes and cost-effectiveness, the limitation is the inability to directly assess and control for confounding factors or perform individual-level analyses and to capture important covariates or environmental factors that could confound genetic associations. |
|  | b) | Mechanism: Discuss underlying biological mechanisms that could drive a potential causal relationship between the investigated exposure and the outcome, and whether the gene-environment equivalence assumption is reasonable. Use causal language carefully, clarifying that IV estimates may provide causal effects only under certain assumptions | 6-9 | The interaction between genetic predispositions and environmental or lifestyle factors is a critical aspect of the pathogenesis of PD. While this study focuses on elucidating the genetic basis of PD through MR analyses, incorporating environmental and lifestyle factors into future analyses is essential for constructing a comprehensive risk model for the disease. |
|  | c) | Clinical relevance: Discuss whether the results have clinical or public policy relevance, and to what extent they inform effect sizes of possible interventions | 9-10 | The identification of immune cell phenotypes associated with PD risk can facilitate patient stratification in clinical trials. Stratifying patients based on their immune cell profiles may help identify subgroups that are more likely to benefit from targeted interventions, thereby enhancing treatment efficacy and patient outcomes |
| 17 | **Generalizability** | Discuss the generalizability of the study results (a) to other populations, (b) across other exposure periods/timings, and (c) across other levels of exposure | 8 | Firstly, given that this study relies on data from a European-origin population, it's important to consider the potential impact of population stratification on the observed associations. European populations are genetically diverse, and differences in allele frequencies can exist between subpopulations, such as those from different regions or with different ancestries. Considering the genetic heterogeneity among different ethnic groups, the results may vary from population to population. Therefore, whether our findings can be generalized to other ethnic groups needs to be further verified by prospective studies. |
|  | **OTHER INFORMATION** |  |  |  |
| 18 | **Funding** | Describe sources of funding and the role of funders in the present study and, if applicable, sources of funding for the databases and original study or studies on which the present study is based | 10 | This research was funded by the National Natural Science Foundation of China (grant number 62171077) |
| 19 | **Data and data sharing** | Provide the data used to perform all analyses or report where and how the data can be accessed, and reference these sources in the article. Provide the statistical code needed to reproduce the results in the article, or report whether the code is publicly accessible and if so, where | 10 | The original contributions presented in the study are included in the article/Supplementary Material. |
| 20 | **Conflicts of Interest** | All authors should declare all potential conflicts of interest | 10 | The authors declare no conflict of interest. |

This checklist is copyrighted by the Equator Network under the Creative Commons Attribution 3.0 Unported (CC BY 3.0) license.

1. Skrivankova VW, Richmond RC, Woolf BAR, Yarmolinsky J, Davies NM, Swanson SA, et al. Strengthening the Reporting of Observational Studies in Epidemiology using Mendelian Randomization (STROBE-MR) Statement. JAMA. 2021;under review.

2. Skrivankova VW, Richmond RC, Woolf BAR, Davies NM, Swanson SA, VanderWeele TJ, et al. Strengthening the Reporting of Observational Studies in Epidemiology using Mendelian Randomisation (STROBE-MR): Explanation and Elaboration. BMJ. 2021;375:n2233.
